# Evaluation of the Stability of Coated Plates with Antigen at Different Temperatures and Times by ELISA Test to Diagnose Fasciolosis

**Published:** 2010-03

**Authors:** MB Rokni, M Aryaeipour, S Koosha, MT Rahimi

**Affiliations:** 1 Department of Parasitology and Mycology, School of Public Health, Tehran University of Medical Sciences, Tehran, Iran; 2 Parasitology and Mycology Division, Laboratory Sciences Department, Paramedical School, Shaheed Beheshti University M.C., Tehran, Iran

**Keywords:** Fasciolosis, ELISA, Plate, Validity, Sensitivity, Specificity

## Abstract

**Background:**

Considering that ELISA method presently is the test of choice for diagnosis of fasciolosis, the present study was undertaken to evaluate the maximum validity of coated plates at different temperatures and different times during one year of evaluation.

**Methods:**

Serum samples of patients infected with fasciolosis (n=10), hydatidosis (n=5), toxocariasis (n=5), and negative control sera (n=5) were examined. Two series of plates were considered. The first series were coated with *Fasciola* homogenate Ag 12 ug/ml, and after some steps were blocked with gelatin and preserved at different temperatures as −80°C, −20°C, −4°C and +4°C. The 2^nd^ series were treated under the same criteria but were not blocked with gelatin. Each series were examined by ELISA test from 1^st^ month to 12^th^ month. Sera with 1:125 dilution, and peroxidase-conjugated goat anti-human IgG diluted 1:10000 were considered optimum.

**Results:**

To ease reporting the results and due to many similarities only results related to 1^st^, 6^th^ and 12^th^ months were analyzed and sensitivity, specificity plus cut-off were determined for each series separately.

**Conclusion:**

Preserving the coated plates, while unblocked at −80°C for 6–8 months is pertinent and functional and in that case, we can be sure the best out put would be applicable.

## Introduction

Fasciolosis, caused by liver fluke species of the genus *Fasciola*, i.e. *F. hepatica and F. gigantica*, is a cosmopolitan parasitic disease (1). The life cycle of the parasite is such that after ingesting metacercaria from contaminated plants and after 3 months, the parasite establishes itself in the biliary ducts of the liver and the infected patient starts to release parasites’ eggs through the faeces (2). It is possible that a low portion of the parasites loss their way to the liver and settle in other parts of the body, which in this case are called ectoparasite (1).When looking at the parasite life cycle, in the first glance, it would be logical to assume that diagnosis of fasciolosis via stool exam and finding parasite's egg would at the frontier line of investigation, but instead, and as it has been attested by many researchers so far, this kind of diagnosis is of low sensitivity, mostly because of scanty of egg releasing, need to special stool exam methods, the possibility of transit infection and so on (3, 4).

Considering the aforementioned fact utilizing immunological and serological diagnostic methods is confirmed by nearly all researchers and all the obstacles mentioned earlier would have no place in these methods. Besides, the sensitivity and specificity of these methods are acceptable and totally show a rate of 92–100% and 84–100%, respectively (4–7). Obviously, among all evaluated methods ELISA should be placed at the top of the scale. This test has demonstrated itself as an authentic, sensitive, and specific method in diagnosing of fasciolosis (4, 7–9).

Considering that ELISA method presently is the test of choice for diagnosis of fasciolosis, the present study was undertaken to evaluate the maximum validity of coated plates with *Fasciola* antigen at different temperatures and different times during one year of evaluation. No doubt, the output could be constructive for other parasitoses, regardless of fasciolosis.

## Materials and Methods

### Antigen preparation

Adults *Fasciola* like worms were obtained from infected sheep livers collected from local abattoirs and were washed 6 times using PBS/ pH 7.2. Somatic antigen was prepared by homogenizing adult worms in 0.045 M PBS/pH 7.2 using electrical homogenizator (Edmund Buhler Co., model Homo 4/A mit uhr) followed by sonication (Tommy Seiko model UP-200P, Tokyo), and then centrifugation at 15000g at 4° C for 30 min. The supernatant was filtered and stored at −20°C until further exploit.

### Clinical Sera

Blood samples were collected from individuals infected with *Fasciola* spp*.* (n=10) from Gilan Province, northern Iran, diagnosed based on stool examination and ELISA test. Informed consent was taken from each patient. Serum samples of patients infected with hydatidosis (n=5), toxocariasis (n=5), and negative control sera (n=5) were examined as well. The latter diseases were diagnosed at the Dept. Medical Parasitology and Mycology, School of Public Health, Tehran university of Medical Sciences, Iran using a variety of diagnostic methods.

### ELISA

Two series of plates were considered. The first series were coated with *Fasciola* antigen, and after some steps were blocked with gelatin and preserved at different temperatures as −80°C, −20°C, −4°C and +4°C.

The 2^nd^ series were treated under the same criteria but were not blocked with gelatin. Each series were examined by ELISA test from 1^st^ month to 12^th^ month. The immunodiagnostic assay was performed as previously described (8), with some modifications. The optimum conditions for ELISA obtained as follows: antigen 12 µg/ml, sera 1:125 dilution, and peroxidase-conjugated goat anti-human IgG diluted 1:10000. Afterwards, 100 microliters of antigen was dispensed into the wells of microtiter plates (Nuclon, Kamstrup, Roskilde, Denmark) and incubated at 37°C overnight. Excess binding sites were blocked with 200µl of bovine serum albumin (2% diluted in PBS/ 0.1% Tween 20) and incubated for 30 min at 37°C. For non-blocked series, the latter step was omitted. After the wells were washed three times with PBS/ Tween 20, 100 µl of a serum sample was added to each plate and incubated for 30 min at 37 °C. Following another washing step, 100 µl of per-oxidase-conjugated goat anti-human IgG (Sigma Chemical Co., Poole, Dorset, United Kingdom) was added to each well and the plates incubated for a further 30 min at 37°C. Following a final washing step, 100 µl of O-phenylendiamine dihydrochloride (OPD) substrate (Sigma Chemical Co., Poole, Dorset, United Kingdom) was added to each well and the reaction stopped after 5 min by adding 50 µl of 12.5% H2S04. The optical density (OD) of the samples was measured at 492 nm using a Titerteck (Helsinki, Finland) multiscan ELISA plate reader. All assays were tested in triplicate and repeated twice.

### Statistical analysis

Cut-off value was calculated as mean plus 3.09 standard deviation OD value of the healthy group sera. Accordingly, OD values more than it were considered as positive and vice versa. The sensitivity, specificity, positive and negative predictive values were calculated using the method of Galen (10). Statistical analysis was carried out using SPSS for Windows, version 11.

## Results

To make easier reporting the results and due to many similarities only results related to 1^st^, 6^th^ and 12^th^ months were analyzed and sensitivity, specificity plus cut-off were determined for each series separately.

Tables 1 and 2 show the sensitivity, specificity, and cut-off values for coated and blocked plates (Table 1) and non-blocked (Table 2) at different temperatures and different times during 1^st^, 6^th^ and 12^th^ month. Accordingly, maintenance of non-blocked plates at −80°C with sensitivity and specificity of 100% and 93.7% after 6 months vs. 91% and 88% of same values in terms of time and temperature for blocked plates showed the ideal conditions for this purpose.

**Table 1:** Sensitivity, specificity, and cut-off values for coated and blocked plates at different temperatures during 1^st^, 6^th^ and 12^th^ month

Temperature (°C)	Sensitivity (%)			Specificity (%)			Cut-off		
	
	1^st^ month	6^th^ month	12^th^ month	1^st^ month	6^th^ month	12^th^ month	1^st^ month	6^th^ month	12^th^ month
−80	100	91	100	93.7	88	83	0.43	0.36	0.12
−20	100	91	91	93.7	88	88	0.40	0.38	0.25
−4	100	83.3	91	88	78.9	75	0.48	0.29	0.15
+4	100	83.3	91	88	83	83	0.60	0.32	0.14

**Table 2:** Sensitivity, specificity, and cut-off values for coated and non-blocked plates at different temperatures during 1^st^, 6^th^ and 12^th^ month

Temperature (°C)	Sensitivity (%)			Specificity (%)			Cut-off		
	
	1^st^ month	6^th^ month	12^th^ month	1^st^ month	6^th^ month	12^th^ month	1^st^ month	6^th^ month	12^th^ month
−80	100	100	91	93.7	93.7	88	0.46	0.35	0.14
−20	100	91	91	93.7	88	83	0.49	0.41	0.24
−4	100	83.3	83	88	88	83	0.45	0.39	0.32
+4	100	91	76.9	88	83	78.9	0.42	0.31	0.15

## Discussion

In defiance of using ELISA as a reliable method to diagnose parasitic disease, the stability of coated plates, whether blocked or unblocked, has been perpetually contentious in debates on validity of the test at different temperatures and different times. Nowadays for many logical reasons time is of the essence, hence every lab authority is perpetually looking to make the time of the test as less as possible. Researchers have focused mostly on improving the method of ELISA due to its simplicity and functionality in diagnosis of parasitic diseases (3, 4 8, 11–13).

In our laboratory as the reference lab for diagnosis fasciolosis and hydatidosis, these questions always have been arising as to what extent a coated plate with antigen can be stored, what temperature is the best for preserving the coated plates, is it better to block plates first and then store them for a long time or block them immediately before any test while storing the plates, and as such. Surveying literature yielded not too much useful data in this topic, so we decided to challenge this issue according to a well-designed project, as already was mentioned in Materials and Methods section.

Coating a plate needs an overnight incubation period and this time can be economized on storing the plates. Nevertheless, the more important factor is that preparing a fresh and efficient always is of high concern for each person involving in doing ELISA test. Preparing antigen is a time-consuming process and most of the times after a lot of work the output is unsatisfied, so if we can a device a manner, through which, we can sore the coated plates for along time, this issue would no more be annoying.

In a bird's eye view, the results of our study showed that storing coated plates for a long time would be applicable in terms of validity of the outputs (Table 1, 2). However, it would be better if the plates were left unblocked until the time of executing the test (Table 2). According to obtained results, maintenance of non-blocked plates at −80°C with sensitivity and specificity of 100% and 93.7% after 6 months vs. 91% and 88% of sensitivity and specificity for same criteria of time and temperature for blocked plates demonstrated that it would be applicable if we want to keep the coated plates for a long time. Even when we go further in time and analyses the outputs in terms of sensitivity and specificity, we notice that obtaining 100% and 83% sensitivity and specificity for blocked plates after 12 months and 91% as well as 88% as to non-blocked plates with similar situation allow us to keep the coated plates even for longer times.

In conclusion, preserving the coated plates, while unblocked at −80°C for 6–8 months is pertinent and functional and in that case, we can be sure the best output would be applicable. We believe that the examined samples are not enough to judge openly as to the validity of the test but considering our objective it would acceptable. A convenient idea would be evaluating this method using more number of sera from patients infected with different parasitoses.
